# The Effect of Silanized Halloysite Nanotubes on the Structure of Polyethylene–Based Composite

**DOI:** 10.3390/ma17133260

**Published:** 2024-07-02

**Authors:** Martina Wieczorek, Tetiana Tatarchuk, Katarzyna Skórczewska, Joanna Szulc, Jolanta Tomaszewska

**Affiliations:** 1 Faculty of Chemical Technology and Engineering, Bydgoszcz University of Science and Technology, 85326 Bydgoszcz, Poland; marwie004@pbs.edu.pl (M.W.); joanna.szulc@pbs.edu.pl (J.S.); 2 Faculty of Chemistry, Jagiellonian University, 30387 Kraków, Poland; tatarchuk.tetyana@gmail.com; 3 Educational and Scientific Center of Materials Science and Nanotechnology, Vasyl Stefanyk Precarpathian National University, 76018 Ivano-Frankivsk, Ukraine

**Keywords:** halloysite, high–density polyethylene, silanization, structure properties

## Abstract

Chemical modification of the surface of halloysite nanotubes (HNT) by alkalization (with sodium hydroxide (NaOH)) and grafting with silanes (bis(trimethylsilyl)amine (HMDS)) was carried out. The efficiency of the alkalization and grafting process was evaluated by X–ray diffraction (XRD), Fourier–transform infrared spectroscopy (FTIR), scanning electron microscopy (SEM), and the nitrogen adsorption method were used. XRD and FTIR analysis confirmed the formation of bonds of trimethylsilyl groups to the HNT surface which changed the nature of the surface from hydrophilic to hydrophobic. In addition, it was noted that grafting with silanes decreases by 7.2% the specific surface area of the halloysite compared to the alkalized material. High–density polyethylene (HDPE) composites with halloysite (HNT), alkalized halloysite (alk–HNT), and HMDS–modified halloysite (m–HNT) were processed in the molten state in a Brabender mixer chamber. On SEM/EDS micrographs of HDPE composites with silanized HNT, a change in surface characteristics from smooth to ductile was observed. Higher melting point values based on differential scanning calorimetry (DSC) analysis of HDPE composites with 5%wt silanized halloysite in comparison with HNT and alk–HNT of, respectively, 2.2% and 1.4% were found, which indicates a slight beneficial influence of the filler on the quality of ordering of the crystalline phase of the matrix.

## 1. Introduction

The polymer plastics industry is continuously growing; in 2022, global production of these materials amounted to 400.3 million tons, of which polyolefins accounted for more than 45%. In terms of the volume of consumption, this group of plastics is dominated by different varieties of polyethylenes, which are widely used in the packaging, automotive, construction, and everyday objects industries [1]. Such ubiquity of applications implies that despite efforts to segregate post–consumer waste, environmental pollution with these plastics continues to grow. There is a growing need for effective waste management, especially of polyethylene, so intensive research on recycling and recovery of this material is being conducted [2,3,4]. Research on the production of new composite plastics using recycled polyethylene in combination with natural fillers is important in terms of environmental performance [5,6,7]. Particular research attention is focused on the production of bio–based packaging, which contains natural fillers in addition to polymers [8,9]. Materials research is relevant to the potential use of high–density polyethylene (HDPE) with natural fillers as materials in the seafood packaging industry with beneficial properties [10,11].

The most important factors affecting the properties of both recycled and virgin pellet matrix composites include origin, aspect ratio, size, and filler content [12]. The interfacial interactions between the filler and the polymer and the degree of dispersion of the filler in the polymer matrix are also relevant. The main problem of manufacturing polyolefin matrix composites with natural fillers is the compatibility between the hydrophobic polymer matrix and the highly hydrophilic surface of natural fillers [13,14,15]. In this connection, research is continuing to develop easily dispersible inorganic compounds or to modify the surface of commonly used fillers [16].

In order to improve interactions at the polyethylene–filler interface, compatibilizers that lead to favorable dispersion of particles in the matrix are used [17,18]. Singh et al. [19] found that the introduction of a compatibilizer such as maleic anhydride grafted with polyethylene into HDPE composites with halloysite nanotubes (HNTs) contributes to an increase in interfacial adhesion, which favorably increases thermal stability. The strengthening effect was negligible in that case, which was explained by the deformation of HNTs due to shear forces during processing.

Halloysite is a double–layered mineral belonging to the kaolin group; it has gained recognition due to its large specific surface area, free spaces inside the structure, and the differentiated structure of the individual layers. Its distinctive property is the presence of water between adjacent tetrahedral and octahedral layers. Halloysite may be found as plates, tubes, and/or mixed structures. The outer surface of halloysite consists of Si–O–Si siloxane groups and Si–OH silanol groups, while the inner surface is made up of Al–OH groups [20,21,22,23]. The material generally shows good dispersion in the polymer matrix, even in an unmodified form, but when at higher concentrations (>10% by weight), it can form agglomerates [24,25]. Another major limitation of halloysite applications involves insufficient adhesion between the outer, negatively charged surface of the nanotubes and non–polar polymers, especially polyolefins, which can, however, be improved by modifying the mineral surface or using compatibilizers [26,27].

One effective method for chemical modification of the external surface of HNTs involves the application of silane coupling agents [28,29]. A two–step method is also used, involving initial activation of the surface with acids/alkalis to eliminate impurities and increase the specific surface area of the material [30,31,32], followed in the second step, i.e., grafting with silanes [12,33]. Sun et al. [34,35] found that the activation of the surface of halloysite nanotubes with acids and alkalis prior to grafting with silanes contributed to increased reactivity of their surface, resulting in increased strength and thermal resistance of epoxy resins containing modified HNTs. In contrast, physical modification of halloysite leads to van der Waals interactions, hydrogen bonds, or electrostatic forces with the polymer matrix. Wang et al. [36,37] carried out a physical modification method for bamboo fibers through impregnation with inorganic CaCO_3_ nanoparticles. They found that impregnating the fibers was an effective way to improve the interfacial interactions between them and the HDPE matrix, which resulted in improved tensile properties. Carrera et al. [38], for their part, used polyvinyl alcohol–modified montmorillonite as a filler for HDPE films. They reported improved thermal properties and reduced CO_2_ permeability compared to pure HDPE.

The use of halloysite nanotubes as HDPE fillers has been reported in the literature [19,39,40,41], with unmodified filler being the most commonly used. Despite the presence of numerous HNT agglomerates in the matrix, an increase in the elastic modulus [41] and heat resistance of the polymer was found [40]. In addition, a recent literature report indicates the potential application of HNTs for packaging production [42].

There are many reports regarding surface modification of halloysite nanotubes by alkalization [43,44,45] and silanization using vinyltrimethoxysilane [46], (N,N–dimethylaminopropyl)trimethoxysilane [47], 3–aminopropyltriethoxysilane [48], (3–glycidyloxypropyl) trimethoxysilane [49], 3–methacryloxypropyltrimethoxy [50], and (3–Mercaptopropyl) trimethoxy silane [51]. Studies where alkalization and silanization are used simultaneously using sodium hydroxide and 2–(3,4–epoxycyclohexyl) ethyltriethoxysilane [52] are also available. On the other hand, hexamethyldisilazane (HMDS) was used for surface modification of anodic aluminum oxide [53], nanoclay [54], and organoclays [55].

In contrast, the two–step halloysite surface modification by alkalization and grafting with hexamethyldisilazane and the use of functionalized material as a filler for waste–derived high–density polyethylene have not yet been described. The expected result of the modification of HNTs is to obtain a favorable structure and, consequently, functional properties of HDPE composites associated with a homogeneous dispersion and increase in the interactions between polymer and filler. The effectiveness of modified HNTs as high–density polyethylene nanofillers was evaluated based on structure studies of HDPE composites. Comparative studies of polyethylene composites modified with alkalized halloysite were also carried out.

## 2. Materials and Methods

### 2.1. Materials

High–density polyethylene (HDPE) Tipelin BA 550–13 by Tiszai Vegyi Kombinát Nyrt (Tiszaujvaros, Hungary) from the waste of thin–walled pipes generated in the milling (MFR_190 °C/21 kg/8/2_ = 39.2 g/10 min, density 0.946 g/cm^3^) was used to prepare the composites. As the filler, halloysite nanoclays (HNT) (linear pattern: Al_2_Si_2_O_5_(OH)_4_·2H_2_O) purchased from Merck (Darmstadt, Germany), (CAS number 1332–58–7) with a molecular weight of 294.19 g/mol were used. Hexamethyldisilazane (HMDS), ([(CH_3_)_3_Si]_2_NH) with 99% purity and Mw = 161.39 g/mol (CAS Number 999–97–3), purchased from Merck (Germany), sodium hydroxide (NaOH) and toluene (C_6_H_5_CH_3_) purchased from Sfere Sim (Lviv, Ukraine) were used for HNT modification. All of the chemical reagents were used without further purification.

### 2.2. HNTs Modification

#### 2.2.1. Alkali Treatment

Sodium hydroxide is a commonly used alkali for surface hydroxylation and thinning the walls of HNTs. The HNTs (50 g) were dispersed in distilled water (410 mL) and NaOH (0.24 g) was added to the suspension. The mixture was stirred with a magnetic stirrer for 5 h at room temperature. The resulting solid phase of alkali–treated HNTs was then filtered and washed several times with water to pH 7 and separated by centrifugation. The prepared alk–HNT was dried at 110 °C for 2 h.

#### 2.2.2. Modification of HNTs by Hexamethyldisilazane (HMDS)

**Figure 1:**
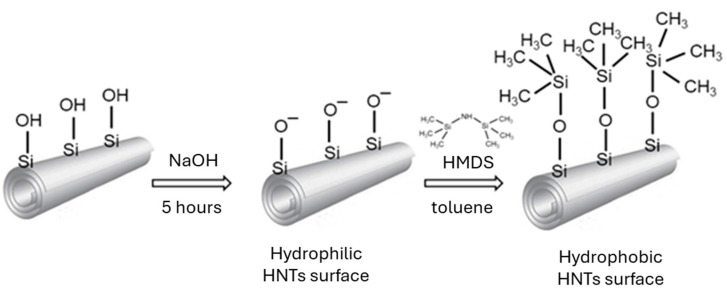
Scheme of possible hydrophobic surface modification of the HNTs through hexamethyl–disilazane (HMDS) grafting.

The grafting of hexamethyldisilazane to alk–HNTs’ surface was conducted following the procedure. First, 5 mL of HMDS was dispersed in 100 mL of toluene for 1 h. The 17 g alk–HNT sample was added to the HMDS/toluene solution and suspension was refluxed at 60 °C for 4 h under constant stirring and 4 h without heating. Toluene was chosen because it is the best solvent according to [51]. The modified powder was filtered and washed with toluene a few times to remove the HMDS excess. Then the hexamethyldisilazane–modified HNTs were dried for 2 h at 110 °C to remove surface–adsorbed moisture. The HMDS amount, necessary for uniform distribution onto the HNTs’ surface, was calculated according to Equation (1):

m(HMDS) [g] = m(HNTs) × S(HNTs)/SSW(HMDS)(1)

where m(HMDS) is mass of HMDS, g; m(HNTs) is mass of HNTs, g; S(HNTs) is surface area of HNTs, S = 64 m^2^g^−1^ (according to Sigma–Aldrich, St. Louis, MO, USA); SSW(HMDS) is specific wetting surface of HMDS, SSW = 485 m^2^g^−1^. The scheme of hydrophobic surface modification of the HNTs through hexamethyldisilazane (HMDS) grifting is depicted in Figure 1.

### 2.3. Characterization Methods of HNTs and Modified HNTs

X–ray patterns were recorded on a STOE STADI P diffractometer (Darmstadt, Germany) with a Cu kα anode (λ = 0.154 nm) in the range of angles 2θ = 4–100°. Determination of the peak positions on the diffraction patterns was performed using Rietveld analysis. The X–ray diffraction allows us to determine the phase composition of crystalline samples.

The specific surface area was determined by the multipoint Brunauer–Emmett–Teller method (BET) using Micromeritics’ Gemini VI instrument. Samples were desorbed at 110 °C for 8 h in helium flow before measurement.

Infrared spectra (IR) were recorded with a Specord M80 spectrophotometer (Carl Zeiss, Oberkochen, Germany). The samples were ground with KBr in a 1:100 ratio and pressed into pellets. The IR spectra were recorded in the frequency range 400–4000 cm^−1^ (number of scans: 64).

Air–dried, uncoated samples were examined by scanning electron microscopy (SEM) using a variable pressure field emission scanning electron microscope (FEI Quanta 200).

### 2.4. Preparation of HDPE/HNT, HDPE/alk–HNT, HDPE/m–HNT Composites

Halloysite powders were kept in a vacuum oven at 80 °C for 24 h to remove absorbed moisture before mixing. Mixtures of polyethylene successively with the halloysite (HDPE/HNT), the alkalized halloysite (HDPE/alk–HNT), and the HMDS–modified halloysite (HDPE/m–HNT) were prepared by mixing with the use of a high shear mixer. The weight percentage of fillers in HDPE mixtures was 1 wt%, 3 wt%, and 5 wt%. The mixtures were processed in the plasticized state by kneading in the chamber of a Brabender FDO 234H type plastographometer with a wall temperature of 185 °C, using a rotation speed of the main rotor of 30 min^–1^, with a friction of 1:1.5. The kneading duration was 15 min. Unfilled HDPE was processed under identical conditions.

The plasticized materials were pressed at 185 °C at a pressure of 20 MPa using a hydraulic press to obtain moldings of 100 mm × 100 mm in size with a thickness of 2 and 4 mm, which were subjected to colorimetric tests after visual evaluation. The composition of the mixtures and their determinations are shown in Table 1.

### 2.5. Testing Methods of HDPE/HNTs Composites

The structure of prepared composites was determined using Fourier–transform infrared spectroscopy (FTIR). The study was carried out using an Alpha apparatus from Bruker, by the ATR (reflective) technique, in the range of 4000–500 cm^−1^, 40 scans at a resolution of 4 cm^−1^ were applied.

The morphology of prepared composites was characterized by a scanning electron microscope Quanta FEG 250 (FEI) in low vacuum conditions at a pressure of 70 Pa with a beam energy of 10 keV. EDS analysis was realized with an electron beam energy of 20 keV using an EDS Octane SDD detector (EDAX).

Determination of the color of the composites was carried out with a Chroma Meter CR–410 Konica Minolta (Japan) colorimeter using a D65 light source, a 10° observer on the CIE L*a*b* scale. On this scale, L* denotes the brightness (0–100), a* the proportion of red/green color (−150–150), and b* the proportion of yellow/blue color (−150–150). The color measurement of each sample was repeated 3 times.

From the color parameters L*a*b*, the color differences of the obtained HDPE/HNTs composites were determined using the following formula:

(2) $$∆E=\sqrt{{{(∆L}^{*})}^{2}+{{(∆a}^{*})}^{2}+{{(∆b}^{*})}^{2}}$$

where ΔE is the color difference; L*, a* and b* are color parameters.

The color interpretation was based on the following ranges of ΔE values [56]:

ΔE < 1—unrecognizable difference;

1 < ΔE < 2—difference recognizable only by an experienced observer;

2 < ΔE < 3.5—difference recognizable by the observer;

3.5 < ΔE < 5—clear color difference;

ΔE > 5—the observer gets the impression of two different colors.

Determined color parameters in the CIELAB system were converted to the RGB color model [57]. In the RGB color model, red, green, and blue primary colors of light are added together in various ways to reproduce a broad array of colors. The RGB color parameters were used to present the effect of filler presence.

The thermal investigations were performed by the differential scanning calorimetry method (DSC) using the Phoenix DSC 204 F1 Netzsch apparatus in standard conditions. Samples of 5–8 mg were heated from 20 to 220 °C at a temperature rate of 5 °C/min.

## 3. Results

### 3.1. Effects of Halloysite Modification

**Figure 2:**
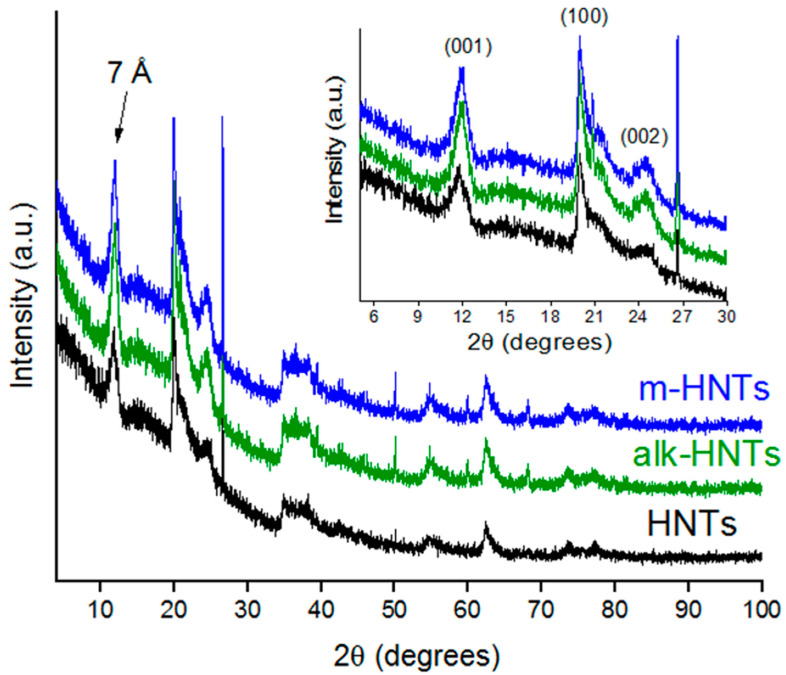
XRD patterns of HNTs before and after alkali treatment and HMDS modification.

The effect of alkali treatment and surface modification on the structure of HNTs was analyzed by XRD. The XRD patterns for raw HNTs, alkali–activated HNTs, and HNTs/HMDS are depicted in Figure 2. According to X–ray diffraction analysis, the main phase in raw HNTs is halloysite–(7 Å) Al_2_Si_2_O_5_(OH)_4_ (JCPDS–29–1487) with a hexagonal crystal system. It can be seen from Figure 2 that all the samples exhibit a (001) diffraction peak at 2θ = 12.114°, characteristic of the halloysite–(7 Å) phase. The other main reflections are observed at degrees 2θ = 12.114; 20.073; 24.572; 35.023; 37.934; 54.547; 62.587, which are in good agreement with other studies [58,59]. The parameters of the crystal lattice of raw HNTs are a = 5.133 Å and c = 7.160 Å. The quartz phase (JCPDS–46–1045) was in a trace. After alkali treatment and HMDS modification, the pattern of HNTs does not change significantly. The observed nonsignificant increase in the peak area and intensity on (001), (100), and (002) plain (inset in Figure 2) is the evidence of the bindings of the trimethylsilyl groups to the HNTs’ surface [60]. The average apparent crystalline size (D) has been calculated from the full width at half–maximum (FWHM) of the (001) peak using the Scherrer equation: D = k·λ/[β_1/2_·cosθ], where k is constant (K = 0.9), λ is 1.5406 Å (Cu radiation wavelength), β_1/2_ is the full width at half–maximum (in radians), and θ is the angle at maximum intensity. The calculated average apparent crystalline size of raw HNTs is 5.9 Å, D(alk–HNTs) = 6.5 Å, D(m–HNTs) = 6.6 Å.

**Figure 3:**
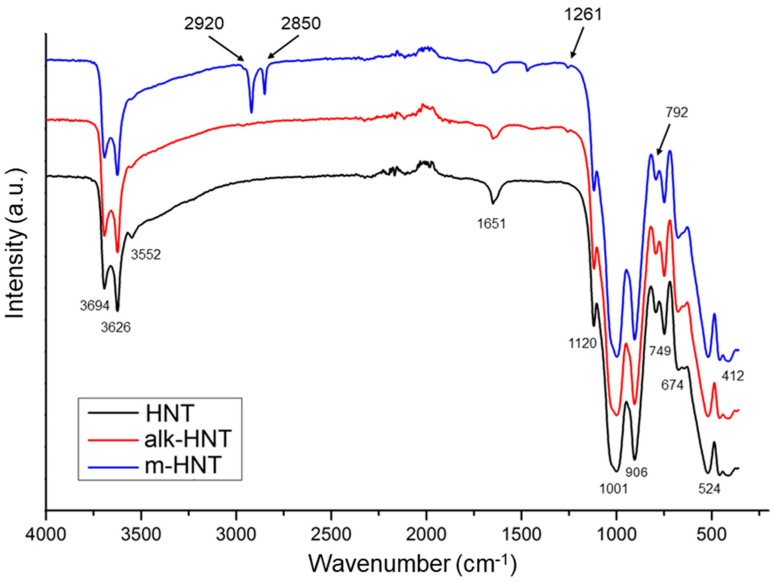
IR–spectra of HNTs surfaces before and after modification.

The IR spectra of all types of HNTs (Figure 3) show the Al_2_O–H stretching absorption bands at 3694 and 3626 cm^−1^, each OH–group being bonded to two aluminum atoms [59]. The peaks observed at 906 and 1001 cm^−1^ were also characteristic of all HNTs and are attributed to the Al–OH and Si–O–Si bonds, respectively [60]. The bands at 792, 749, and 524 cm^−1^ represent the Si–O–Al bonds. The peaks at 674 cm^−1^ and 412 cm^−1^ can be assigned to the Al–OH bonds and Si–O bonds, respectively [61]. The IR spectrum of HNTs modified with HMDS in the presence of toluene as solvent was changed by the appearance of new peaks. The spectrum shows new strong bands for HMDS–modified HNTs at 2920 cm^−1^ and 2850 cm^−1^. They represent the C–H stretching vibrations that occur at 2800–3000 cm^–1^. In the area at 1470–790 cm^−1^ one weak peak appears at 1261 cm^–1^, which corresponds to the Si–CH_3_ symmetric deformation [51,53]. These peaks are evidence of chemical modification of the HNTs’ surface as a result of condensation reaction between the surface of OH–groups onto HNTs and the trimethylsilyl groups of organosilicon modifier. The grafting of the (CH_3_)_3_Si–groups to the HNTs’ surface causes their hydrophobic nature.

The specific surface area of HNTs ranges from 22 to 82 m^2^g^−1^ [21,44,62], and halloysite with specific surface areas of 32 m^2^g^−1^ [63], 65 m^2^g^−1^ [64] and 64 m^2^g^−1^ [65] was introduced into the HDPE matrix. Determined by the low–temperature nitrogen sorption method, the specific surface area of the raw halloysite used in our study was 50.46 m^2^g^−1^. The alkalization carried out before the grafting reaction contributed to increasing the specific surface area of the halloysite to 72.72 m^2^g^−1^. An increase in the specific surface area of halloysite as a result of alkalization with NaOH was observed by White et al. [45]. They found that alkalization for 24 and 84 days contributed to an increase in the initial specific surface area of 24.3 m^2^g^−1^ by 46%, which was explained by a reduction in the thickness of the nanotube walls leading to their disruption and disintegration. Treatment with acid or alkali increases the specific surface area and changes the morphological form of halloysite due to the removal of impurities, and dissolution of HNT structures leading to the increase in the internal diameter of the HNTs’ lumen and surfaces and pores by the roughness treatment, as reported in [66,67].

The introduction of HMDS functional groups results in a reduction in the specific surface area of the halloysite to 67.49 m^2^g^−1^, which confirms an efficient silanization process [31,68]. Similar results were described by Sun et al. [34], who activated the surface of the halloysite with a mixture of H_2_SO_4_ and H_2_O_2_ and then grafted with aminosilanes. The first washing step increased the specific surface area, while the second step associated with silanization led to a more than twofold reduction in specific surface area relative to the alkalized material. Despite the reduction in the specific surface area of HMDS–modified HNTs samples compared to alkali–treated HNTs, BET values are still higher compared to unmodified halloysite, which is beneficial to obtain better interaction between HNTs and HDPE.

**Figure 4:**
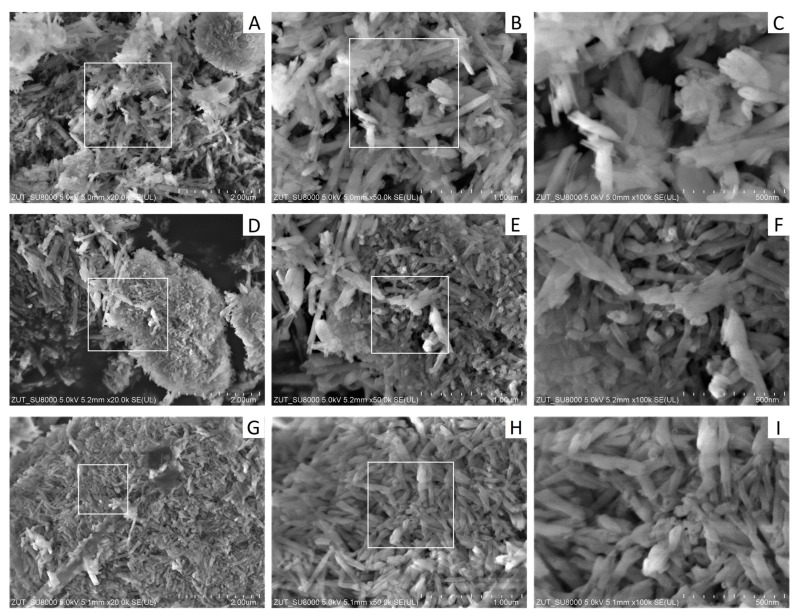
SEM microphotographs of (**A**–**C**) raw halloysite, (**D**–**F**) alkalized halloysite, (**G**–**I**) HMDS–modified halloysite.

The morphology of fillers was characterized by SEM, as shown in Figure 4A–I. The images of raw halloysite (Figure 4A–C) show dispersed halloysite nanotubes, which tend to form small aggregates with a maximum area of 2 µm. The surface of the nanotubes, with outer diameters ranging from 40 to 80 nm and lengths ranging from 200 to 1000 nm, is smooth and homogeneous. Based on SEM microphotographs, similar dimensions and structure have been described in previous articles [15,69,70].

As shown in the image of alkalized halloysite (Figure 4D–F), agglomerates of nanotubes ranging from 3 to 4 µm were observed. The length of the nanotubes did not change while their outer diameter increased and ranged from 60 to 100 nm. A similar effect of changing dimensions of halloysite nanotubes after alkalization reaction was described in previous articles [45,71,72]. Alkaline treatment of the surface of halloysite nanotubes leads to an increase in the inner diameter by reducing the wall thickness, which is not accompanied by a change in its length.

Halloysite nanotubes grafted with silanes are shown on the SEM microphotographs (Figure 4G–I). The dimensions of halloysite nanotubes have decreased, with their length ranging from 200 to 800 nm and outer diameter from 30 to 60 nm. Halloysite nanotubes with a smaller diameter and shorter length were also obtained by Albdiry and Yousif [46], who modified the surface of the nanotubes with vinyltrimethoxysilane without prior surface treatment with acids or alkalis.

As a result of silanization, HNTs have a much more pronounced tendency to form agglomerates (Figure 4G), with the largest agglomerates being about 10 µm in size and occupying the entire surface of the analyzed SEM image. The packing density of HNT aggregates is also higher compared to that of alkali–treated HNTs. A similar tendency to form agglomerates after the silanization reaction was reported by Abu El–Soad et al. [73]. Chen et al. [50] described the beneficial effect of silanization of the surface of halloysite nanotubes while obtaining a material with better homogeneity. Both alkalization and silane modification did not affect the morphological shape of halloysite, while their size and degree of agglomeration changed.

### 3.2. Properties of HDPE/Halloysite Composites

#### 3.2.1. Structure of HDPE/HNTs Composites

**Figure 5:**
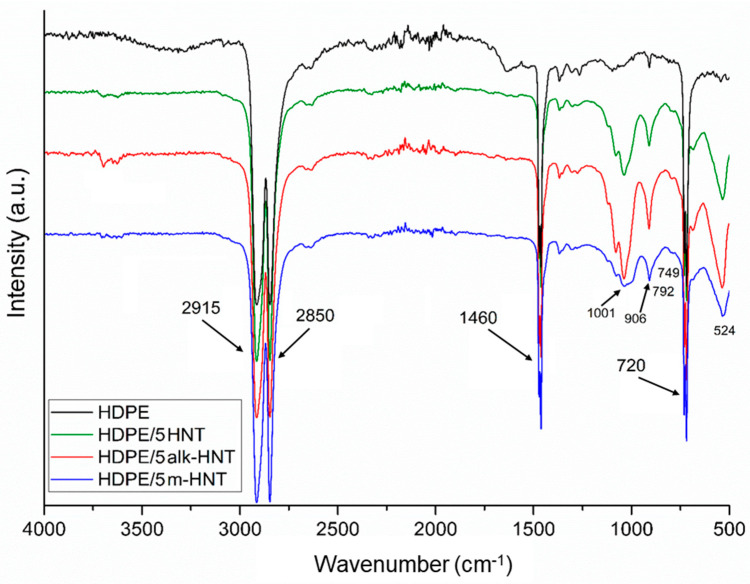
FTIR spectrum of HDPE/5HNT, HDPE/5alk–HNT, HDPE/5m–HNT composites.

The results of the analysis of FTIR spectra of HDPE and HDPE composites with 5 wt% raw halloysite, alkalized halloysite, and modified HMDS are shown in Figure 5. In all spectra, two characteristic bands of high intensity are visible: the first at 2915 cm^−1^, which corresponds to CH_2_ asymmetric stretching and the second at 2850 cm^−1^ originating from CH_2_ symmetric stretching of the methylene group. The next bands at 1460 cm^−1^, which are attributable to CH_2_ deformation vibrations, and at 720 cm^−1^, corresponding to CH_2_ rocking deformation, are also characteristic of high–density polyethylene [74,75,76].

On the FTIR spectra, all the characteristic bands in the range 3600–3700 cm^−1^, 900–1000 cm^−1^, and 524–800 cm^−1^, originating from the fillers introduced into the HDPE matrix, described in the spectra shown in Figure 3, may be found. The intensity of the band in the range 3600–3700 cm^−1^ in the case of the HDPE/5m–HNT sample, however, is lower compared to the intensity of this band on the spectrum of the HDPE/5alk–HNT sample, which may be due to the reaction of silane functional groups originating from unreacted HMDS residue on the nanotube surface under processing conditions, despite repeated washing with toluene. A similar effect was also observed for bands at 1001 cm^−1^ and 906 cm^−1^.

On the spectrum of the HDPE/5m–HNT composite, the presence of bands confirming the modification of the HNT surface by grafting with (CH_3_)_3_ Si–groups is not detectable; the bands at 2920 cm^−1^ and 2850 cm^−1^ that are characteristic of HMDS–modified HNTs, found in Figure 3, are covered by bands in that range originating from polyethylene. However, when Fourier transform infrared spectroscopic method with attenuated total reflectance (FTIR–ATR) is used to evaluate the structure of samples of polymer composites obtained in the plasticized state, a lower intensity or even absence of bands from the filler is often found. The probable reason for this is the composite structure formed under processing conditions, where the filler particles are “embedded” in the melted polymer, and in addition, due to its rheological characteristics in the surface layer of the samples, a skin is formed, in which the polymer is the dominant phase [77,78]. The reflection technique was used to evaluate the structure, so the described effects may be the reason for the absence of the presence of the band recorded for HNTs modified by HMDS at 1261 cm^−1^, evidencing Si–CH_3_ symmetric deformation.

The influence of filler’s presence on the composites’ appearance was observed by measuring color parameters L* (lightness: 0 for black and 100 for white), a* (a* > 0 for red and a* < 0 for green), b* (b* > 0 for yellow and b* < 0 for blue) and ΔE (total color difference).

The colorimetric analysis of the materials is shown in Table 2. The highest value of the L* parameter, and therefore the brightest color, was recorded for HDPE. A gradual increase in the amount of filler in the HDPE matrix, regardless of its type, contributed to a decrease in the L* value, so that the composite was perceived as darker. The highest L* value was observed for HDPE composites with HMDS–modified HNT, while the lowest value was observed for HDPE composites with raw halloysite.

Positive a* and b* values were recorded for both pure HDPE and composites, which translates into red and yellow color of the composites, respectively. With an increase in the proportion of filler in the HDPE polymer matrix regardless of its type, an increase in the values of color coordinates a* and b* is observed, indicating a higher proportion of red and yellow color. The highest values of a* and b* were recorded for the HDPE composite with 5 wt% alkalized halloysite.

Higher values of the L* parameter and, simultaneously, lower values of the a* and b* parameters of pure HDPE were recorded by Mohammadi et al. [79], who conducted colorimetric analysis of HDPE and LDPE films. The prepared films are transparent and achromatic materials accounting for the higher value of the L* parameter and lower values of the a* and b* parameters compared to the HDPE plates analyzed.

The ΔE value of the HDPE plate with the addition of 1 wt% HMDS–modified halloysite was in the range of 2 < ΔE < 3.5, which implies that the color difference is discernible by the observer. The other prepared HDPE composites exhibited a value of ΔE > 5, which implies that the observer gets the impression of two different colors. The HDPE/m–HNT composite plates had the lowest ΔE values, which implies that of the composites tested, they were the ones with the least separation/difference from the unfilled material. The HDPE/HNT composites showed an approximate 44% increase in ΔE values compared to the HDPE/m–HNT composites.

**Figure 6:**
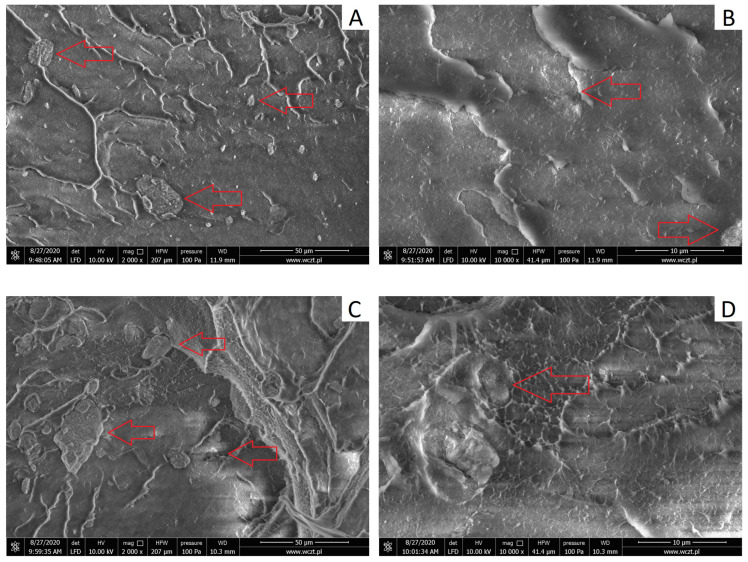

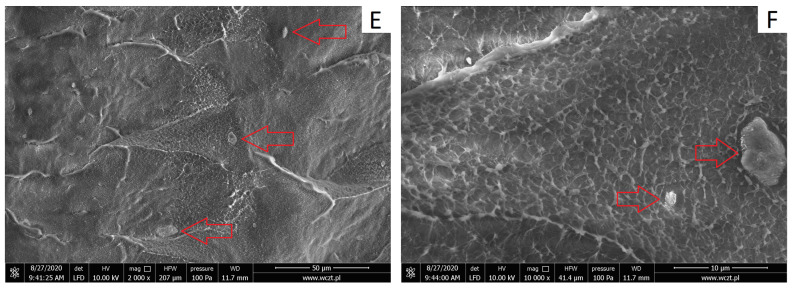
SEM microphotographs of the prepared composites: (**A**,**B**) HDPE/5HNT, (**C**,**D**) HDPE/5alk–HNT, (**E**,**F**) HDPE/5m–HNT.

The morphology of the composites obtained was evaluated by analyzing images from scanning electron microscopy (Figure 6A–F) where red arrows mark the locations of halloysite agglomerates. The fracture surface (Figure 6A,B) of the HDPE/5HNT composite demonstrates the brittle nature of the fracture. Numerous evenly distributed filler agglomerates with a diameter of about 5 µm are observed. Larger filler agglomerates of approx. 25 µm may be seen, which exhibits an uneven surface and shape. Similar observations were reported by Singh et al. [19], who noted the presence of HNT agglomerates in the HDPE matrix at contents greater than 5 wt% by weight. The issue of material aggregation was solved using a compatibilizer that favorably affects the dispersion of the filler in the matrix.

When analyzing the fractures (Figure 6C,D) of the HDPE/5alk–HNT composite, we observe a similar trend of filler agglomeration in the polymer matrix. Numerous agglomerates of approx. 10 µm are not uniformly distributed in the polymer matrix and form larger clusters of up to 40 µm. In the area where the clusters of filler agglomerates occur, a change in the surface to a ductile, irregular one with visible deformation of the polymer matrix was observed. The presence of agglomerates of alkalized halloysite in the polymer matrix is due to the different surface characteristics of the two materials, while the bonding to the polymer matrix is so strong that the filler particles are permanently embedded in the matrix.

A distinct change in the characteristics of the fracture surface was observed for the HDPE/5m–HNT composite (Figure 6E,F). The microphotograph (Figure 6E) shows adjacent areas differing in appearance, i.e., with a flat, smooth surface and a plastic, ductile surface. Modification with silanes contributed to a relatively homogeneous dispersion of the filler in the polymer matrix with single agglomerates with a maximum diameter of approx. 5 µm, which indicates that the processing in the molten state contributed to the reduction in agglomerates of silane–grafted halloysite. Similar observations of surface morphology were described by Chaudhry et al. [80] for HDPE composite with graphite and expanded graphite, with the described effects observed for samples with 20 wt% of filler. In our study, a change in the surface characteristics of HDPE was found with 5 wt% by weight. Different results were presented by Du et al. [81], who noted the presence of numerous clusters of silanized halloysite on the surface of maleic anhydride–grafted polypropylene with stronger adhesion at the polymer–filler interface.

**Figure 7:**
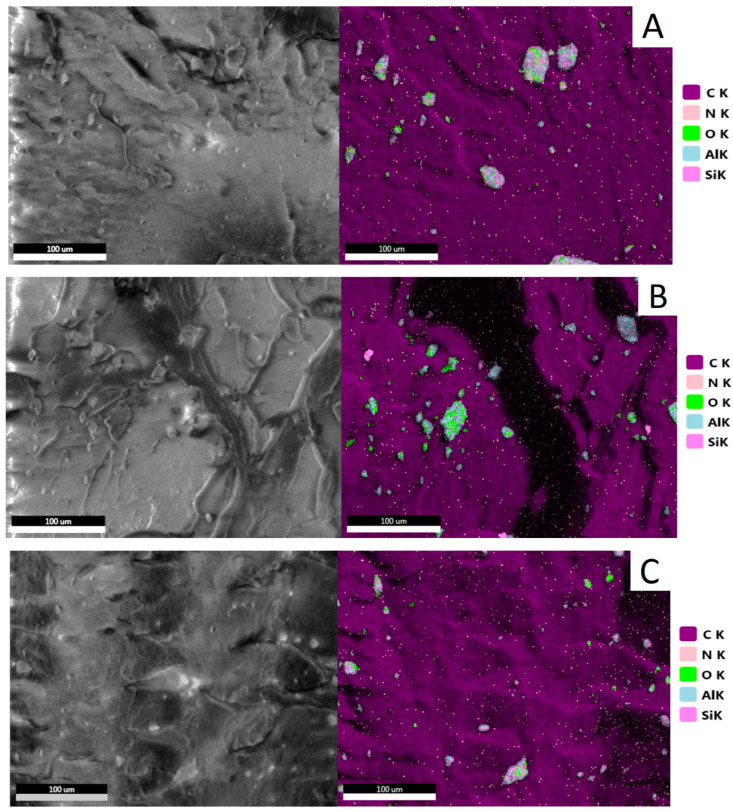
EDS maps: (**A**) HDPE/5HNT, (**B**) HDPE/5alk–HNT, (**C**) HDPE/5m–HNT.

Figure 7 shows SEM/EDS maps of HDPE matrix composites with 5 wt% of fillers. The presence of elements such as carbon, oxygen, silicon, and aluminum was reported and color–coded for identification. The presence of carbon (purple color) is due to the chemical composition of the HDPE polymer matrix. The presence of halloysite (Al_2_Si_2_O_5_(OH)_4_·2H_2_O) agglomerates in the HDPE matrix is indicated by agglomerates of oxygen (green), aluminum (blue), and silicon (pink). The locations of filler agglomerates marked on the SEM/EDS maps are consistent with the SEM microphotographs (Figure 6). The agglomerates with the largest diameter were observed in HDPE/alk–HNT composites. In contrast, single agglomerates of approx. 5 µm were observed on the HDPE/m–HNT composite surface. In addition to the identification of filler agglomerates, regularly occurring, single filler particles are visible across the surface.

**Figure 8:**
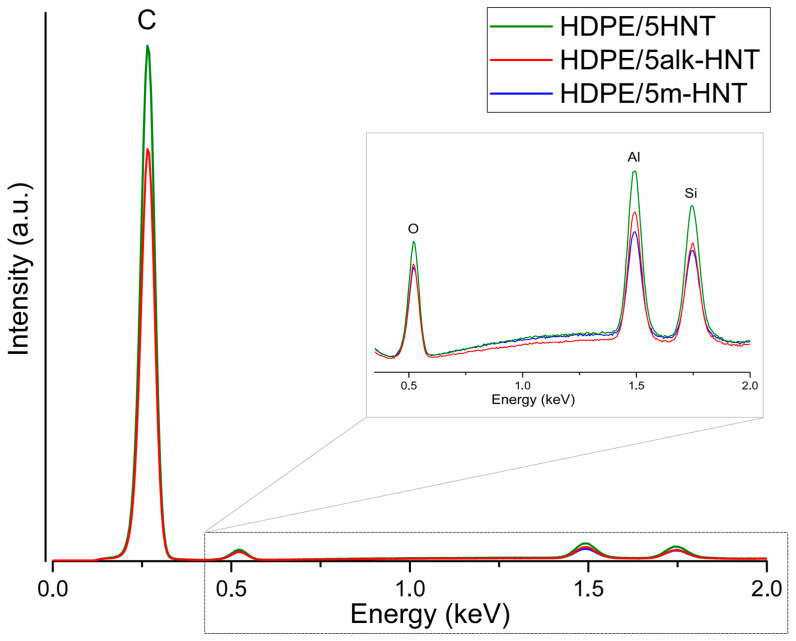
Elemental EDS mapping of composites with 5 wt% raw halloysite, alkalized halloysite, and HMDS–modified halloysite.

The EDS data of the SEM/EDS are shown in Figure 8. The highest intensity of the bands was observed for the composite with raw halloysite, which is consistent with the FTIR analysis (Figure 5), where high–intensity bands associated with the presence of silicon, aluminum, and oxygen were observed at 1001 cm^−1^ and 906 cm^−1^. Both alkalization and HMDS grafting of the halloysite surface resulted in lower band intensities. The lowest intensity of silicon and aluminum bands was noted for HMDS–modified halloysite. The HMDS functional group attached to the halloysite surface contained additional silicon, so the band intensity here should increase. This means that the EDS method is not effective for quantifying the silane content [82,83].

#### 3.2.2. Thermal Properties of HDPE/HNTs Composites

**Figure 9:**
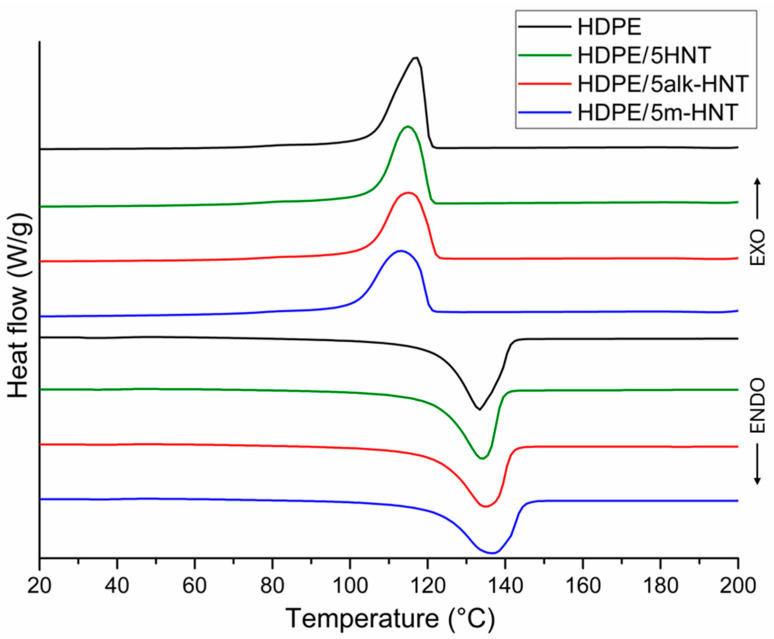
DSC curves of HDPE and HDPE/5HNT, HDPE/5alk–HNT, HDPE/5m–HNT composites.

The thermal history appears in the DSC thermograms when recording the thermal effects occurring during the first heating. Therefore, to evaluate the effect of the filler used, excluding the influence of the temperature conditions of wafer formation, the DSC thermograms obtained during the second heating cycle and second cooling cycle of HDPE samples and selected composite systems containing 5 wt% filler, shown in Figure 9, were used for the analysis. 

Table 3 summarizes the values of melting temperature (T_m_), melting enthalpy (ΔH_m_), crystallization temperature (T_c_), and degree of crystallinity (X_c_) determined from thermograms of unmodified HDPE and HDPE/HNT, HDPE/alk–HNT and HDPE/m–HNT composites. The degree of crystallinity was calculated from the following formula:

X_c_ = ΔH_m_/((1 − w)ΔH^0^_m_) × 100% (3)

where ΔHm is the melting enthalpy of the investigated materials (Jg^−1^), ΔH^0^_m_ is the enthalpy of melting of fully crystalline HDPE of 293 Jg^−1^, and w is the filler content [84].

The degree of crystallinity of unmodified HDPE is 77.6%, while its melting point is 133 °C and its crystallization temperature is approx. 117 °C; all data are consistent with the available literature [84,85].

The introduction of fillers up to 3 wt% does not significantly change the melting point of the composites compared to the T_m_ value of the HDPE sample. The slightly lower melting point of the HDPE/1HNT composite indicates the presence of crystalline structures with poorer ordering compared to those of unfilled HDPE [63].

The increase in melting point found for composites containing 5 wt% filler, regardless of the type, indicates better ordering of the polymer matrix crystallites. The higher T_m_ values of composites containing 5 wt% m–HNT compared to samples containing HNT and alk–HNT may confirm the slight beneficial effect of silane–modified halloysite on the quality of arrangement of the crystalline phase of the matrix and the better rearrangement and more perfect formation of crystals [65] and/or its more favorable dispersion in the matrix. This effect may result in an increase in the packing density of polyethylene macromolecules in the HDPE/5m–HNT composite compared to composites containing the same amount of HNT and alk–HNT fillers.

For all composites except HDPE/1HNT, the crystallization temperature was found to decrease by 0.3 to 3.9 °C compared to the T_c_ value of unfilled HDPE. In addition, the crystallization temperature decreases slightly with increasing filler concentration in the matrix. These results do not confirm the pronounced nucleating effect of HNT described in the literature [70,86]. The decrease in T_c_ with an increase in the proportion of HNTs indicates that the filler may disrupt the formation of crystalline structures of the matrix during its cooling, which indirectly indicates that the dispersion of HNTs is less homogeneous at higher concentrations in the matrix, which was also observed in paper [63].

The degree of crystallinity of unmodified HDPE is 77.6%, and the halloysite fillers used, regardless of type, only slightly affect the degree of crystallinity of the composites, the value of which ranges from 76.3 to 78.3%. The slight increase in the degree of crystallinity of the composites compared to HDPE may suggest that the filler in the smallest amount used in the study is promoting the formation of crystallization nuclei. Gaaz et al. [87] noted that introducing raw halloysite or halloysite modified with urea and imides into the polypropylene matrix (PP) decreases crystallinity, while grafting the surface of halloysite with silanes prior to incorporation into the PP matrix resulted in an increase in the degree of crystallinity. In our case, we found no clear effect of the chemical treatment of halloysite on the degree of crystallinity of the composites; nevertheless, the HDPE/1m–HNT composite had the highest degree of crystallinity.

The literature data indicate that the values of melting and crystallization temperatures, as well as enthalpy and the degree of crystallinity, differ even for HDPE samples from a single source, which is due, in part, to the use of different test parameters, i.e., heating and cooling rates and/or the molecular weight of the polymer and the excipients used [64,88].

The findings of the analysis of thermal properties of low density polyethylene (LDPE) composites with halloysite nanotubes and compatibilizers were presented by Sikora [65], who found a higher crystallization temperature of the composites compared to the pure polymer and, at the same time, lower values of the melting point and enthalpy. In addition, the dependence of the reduction in melting point of the LDPE composite with increasing halloysite content in the polymer matrix can be established. The nucleating effect of halloysite leads to a reduction in the size of polycrystalline aggregates and an increase in the content of the crystalline phase.

## 4. Conclusions

Two–step chemical treatment of halloysite, successively by alkalization leading to an increase in the number of hydroxyl groups on the outer surface of the nanotubes, followed by silanization using hexamethyldisilazane, proved to be an effective modification method in terms of using the material as a filler for high–density polyethylene. The effectiveness of the chemical modification is evidenced by the presence of new strong bands for HMDS–modified HNTs at 2920 cm^−1^ and 2850 cm^−1^ on the FTIR spectra, as well as slight changes in the XRD pattern (increase in surface area and peak intensity on the (001), (100) and (002) surfaces), evidence of binding of trimethylsilyl groups to the HNT surface. The change in the specific surface area of halloysite nanotubes determined by the BET method, which occurred as a result of alkalization and subsequent silanization, is related to the modification of the nanotube surface due to the attachment of hydroxyl and silane functional groups.

HDMS–modified halloysite nanotubes, and for comparison raw and alkalized ones, were introduced into the HDPE matrix by melt blending. Regardless of the type of halloysite used, the presence of filler agglomerates was detected on the fracture surface of the composites based on SEM/EDS observations. In the case of HDPE/5m–HNT composites, a change in character from smooth to ductile was observed; this confirmed the favorable dispersion of halloysite in the HDPE matrix. The brightness and color of HDPE matrix composites differ significantly from the parameters of the unmodified polymer and depend on the type and concentration of the halloysite. The least differences compared to the matrix were found for the HDPE/m–HNT samples, as evidenced by the lowest values of total color difference. Based on composite DSC analysis, it was found that the introduction of 5 wt% halloysite positively affects the quality of ordering of the crystalline phase of the matrix, especially HMDS–modified HNTs.

The research carried out within the scope of this study is an important contribution to the development of knowledge in the field of modification of halloysite nanotubes by silanization and the application of the mineral as a filler for HDPE. The positive effect of halloysite modification by silanization on the structural properties of HDPE matrix composites justifies further research. The potential application of HDPE composites as materials for packaging systems determines the direction of research related to the analysis of mechanical, thermal, barrier and biological properties.

## Figures and Tables

**Table 1:** Composition and determination of high–density polyethylene (HDPE) composites.

Sample	Filler Type	Filler Content, wt%
HDPE		0
HDPE/1HNT	raw halloysite (HNT)	1
HDPE/3HNT		3
HDPE/5HNT		5
HDPE/1alk–HNT	alkalized halloysite	1
HDPE/3alk–HNT		3
HDPE/5alk–HNT		5
HDPE/1m–HNT	Bis(trimethylsilyl)amine (HMDS)–modified halloysite	1
HDPE/3m–HNT		3
HDPE/5m–HNT		5

**Table 2:** Color parameters of plates obtained upon processing.

Sample	L*	a*	b*	R (Red)	G (Green)	B (Blue)	ΔE	Color
HDPE	85.96 (±0.23)	1.30 (±0.13)	2.41 (±0.31)	219	214	210	0.0 (±0.63)	
HDPE/1HNT	81.62 (±0.28)	2.05 (±0.11)	6.43 (±0.24)	211	201	191	5.9 (±0.47)	
HDPE/3HNT	63.44 (±0.41)	3.28 (±0.21)	10.85 (±0.33)	165	151	134	24.0 (±0.55)	
HDPE/5HNT	63.94 (±0.34)	4.66 (±0.19)	13.91 (±0.37)	170	152	130	25.0 (±0.62)	
HDPE/1alk–HNT	83.85 (±0.32)	1.73 (±0.18)	7.62 (±0.19)	217	208	195	5.6 (±0.42)	
HDPE/3alk–HNT	76.81 (±0.27)	4.63 (±0.11)	12.56 (±0.21)	205	186	167	14.0 (±0.29)	
HDPE/5alk–HNT	67.24 (±0.37)	7.89 (±0.14)	14.64 (±0.23)	185	158	138	23.2 (±0.41)	
HDPE/1m–HNT	85.87 (±0.21)	1.41 (±0.16)	4.82 (±0.28)	220	214	206	2.4 (±0.46)	
HDPE/3m–HNT	79.79 (±0.56)	3.19 (±0.18)	11.05 (±0.31)	210	195	177	10.7 (±0.52)	
HDPE/5m–HNT	73.67 (±0.42)	5.67 (±0.20)	14.38 (±0.36)	199	177	155	17.6 (±0.68)	

**Table 3:** Overview of values T_m_, ∆H_m_, T_c_, and X_c_.

Sample	T_m_, °C	ΔH_m_, Jg^−1^	T_c_, °C	X_c_, %
HDPE	133.4 ± 0.2	227.4 ± 0.6	116.9 ± 0.3	77.6 ± 0.3
HDPE/1HNT	132.8 ± 0.3	226.2 ± 0.2	117.7 ± 0.6	78.0 ± 0.2
HDPE/3HNT	134.3 ± 0.6	217.8 ± 0.5	115.7 ± 0.5	76.6 ± 0.3
HDPE/5HNT	134.0 ± 0.5	209.9 ± 0.5	114.8 ± 0.3	76.4 ± 0.2
HDPE/1alk–HNT	134.7 ± 0.2	224.3 ± 0.3	113.0 ± 0.5	77.3 ± 0.6
HDPE/3alk–HNT	134.0 ± 0.3	218.4 ± 0.2	116.6 ± 0.4	76.8 ± 0.5
HDPE/5alk–HNT	135.0 ± 0.4	212.5 ± 0.5	114.8 ± 0.2	76.3 ± 0.4
HDPE/1m–HNT	135.9 ± 0.4	227.5 ± 0.6	114.6 ± 0.5	78.4 ± 0.2
HDPE/3m–HNT	134.0 ± 0.3	221.5 ± 0.4	115.5 ± 0.3	77.9 ± 0.5
HDPE/5m–HNT	136.9 ± 0.6	213.5 ± 0.6	113.0 ± 0.2	76.7 ± 0.3

## Data Availability

The data presented in this study are available on request from the corresponding authors.
